# A case of sialodochitis fibrinosa with MR findings leading to selection of an appropriate treatment

**DOI:** 10.1016/j.radcr.2024.07.104

**Published:** 2024-08-07

**Authors:** Aya Inoue, Koshi Ikeda, Tazuko Goto, Kazuhiko Hashimoto

**Affiliations:** a Department of Oral and Maxillofacial Radiology, Tokyo Dental College, 2-9-18, Kandamisaki-cho, Chiyoda-ku, Tokyo 101-0061, Japan; b Department of Radiology, Tokyo Dental College Ichikawa General College, 5-1-13, Sugano, Ichikawa-shi, Chiba 272-0824, Japan; c Department of Medical Examination and Testing, Tokyo Dental College Ichikawa General College, 5-1-13, Sugano, Ichikawa-shi, Chiba 272-0824, Japan

**Keywords:** Sialodochitis fibrinosa, STIR, Kussmaul disease

## Abstract

Sialodochitis fibrinosa is a rare disease characterized by paroxysmal swelling of the salivary glands and discharge of fibrous masses containing eosinophils from the salivary gland orifice. Diagnosis was traditionally based on irregular dilation of the main salivary duct by sialography, but now includes the imaging findings of magnetic resonance imaging (MRI). In the present patient, short TI inversion recovery (STIR) MRI sequence was able to identify Stensen's duct dilation and additionally depict cystic dilation due to stenosis of the orifice and multiple cystic dilations within the parotid gland body. Treatment was performed on each of the lesion sites identified by MRI. The patient was successfully treated with compressive gland massage for lesions within the body of the parotid, and bougienage was performed for stenosis of Stensen's duct orifice, with duct flushing for dilation of Stensen's duct. These findings suggest that MRI could replace sialography and has the advantages of being noninvasive, having a wide observation area, and enabling observation within the glandular body. Here, we report the case of a patient in whom accurate identification of the site of the lesion enabled selection of appropriate treatment for each site.

## Introduction

Sialodochitis fibrinosa was first reported by Kussmaul in 1897 [1]. Diagnostic criteria have been established for many of the salivary gland diseases [2]; however, there are no established diagnostic criteria for sialodochitis fibrinosa. Murakami et al. described the following features: 1) paroxysmal recurrent swelling of the salivary glands; 2) discharge of material containing abundant eosinophils from the main duct; 3) increased blood eosinophils and IgE; 4) concomitant other allergic processes are common; 5) the sialography findings of narrow main duct of salivary gland, indistinct gland shadows, irregular dilation of main salivary duct, leakage of contrast material from main salivary duct, or apparently normal images; and 6) histopathological findings of duct swelling and eosinophilic infiltration [3]. Sialography is considered useful in the diagnosis of sialodochitis fibrinosa. More recent reports mention computed tomography (CT) and magnetic resonance imaging (MRI) findings but none has evaluated the findings in detail. [[4], [5], [6], [7], [8]] As there is no established treatment, patients are generally managed conservatively. Anti-allergic drugs are often the drug of choice due to frequent complication by allergic disease [9].

To the best of our knowledge, there is no previous report of treatment selection based on imaging findings. Therefore, the aim of this study was to evaluate the utility of the short TI inversion recovery (STIR) MRI sequence as an alternative to sialography in the diagnosis and selection of treatment in sialodochitis fibrinosa. Moreover, we report that identifying the site of the lesion may lead to appropriate treatment.

## Case report

**Fig. 1:**
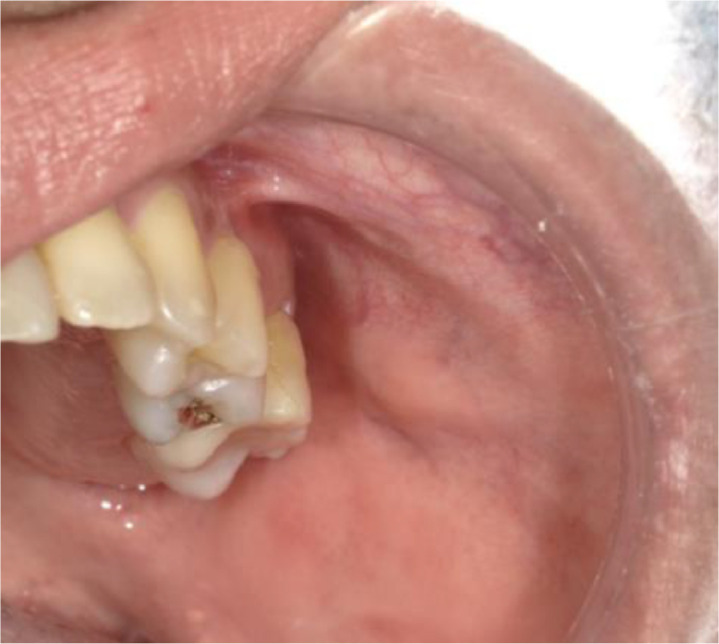
Intraoral photograph at the time of the initial visit. Dilation of the left parotid papilla is apparent.

**Fig. 2:**
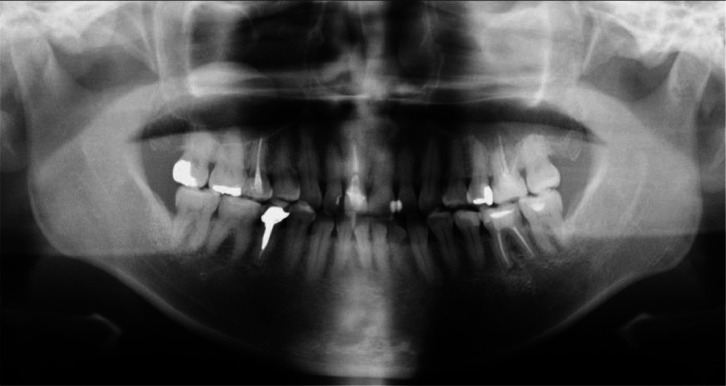
Panoramic radiograph. No sialolithiasis is seen and there are no notable findings.

A 42-year-old woman presented at our hospital complaining of swelling in the left cheek that had discharged a white substance into her mouth for the past several years. Her medical history included asthma, sinusitis, nasal inflammation, gastric ulcer, colitis, duodenal ulcer, anemia, tonsillar abscess, and bipolar disorder. Intraoral examination revealed swelling at the orifice of the left parotid gland. Salivary outflow was not clear (Fig. 1). A panoramic radiograph demonstrated no evidence of sialolithiasis (Fig. 2).

**Fig. 3:**
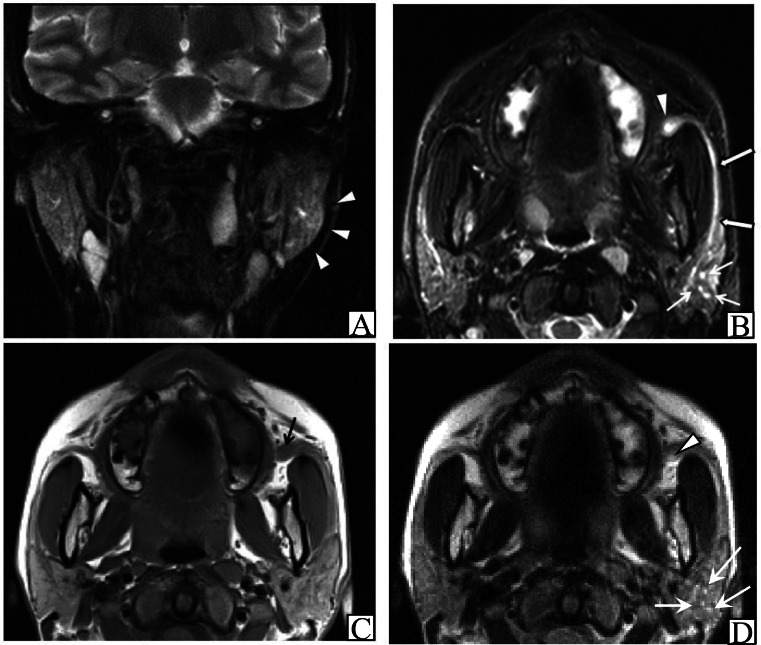
MR images. (A) Coronal STIR image shows obvious swelling of the left parotid gland. (arrowheads). (B) Axial STIR image shows multiple punctate high-signal areas in the body of the left parotid gland (short arrows). Stensen's duct is dilated, partly stenotic, and irregular (long arrows). There is cystic dilation of the orifice due to stenosis near the left Stensen's duct orifice (arrowhead). No opacity is apparent in left-sided periparotid adipose tissue. (C) Axial T1-weighted image shows dilation of the left lateral Stensen's duct orifice (arrow). (D) Axial T2-weighted image reveals multiple spots of high signal intensity within the body of the left parotid gland (arrows). There is high-signal cystic dilation near the orifice (arrowhead).

The left parotid gland was more swollen than the right on STIR imaging (Fig. 3A) and there were multiple cystic dilations within the left parotid gland. The left Stensen's duct was dilated with cystic dilation near its orifice. There was no adipose tissue opacity in the left parotid gland and no thickening of the shallow cervical fascia (Fig. 3B). Dilation of the orifice of the left Stensen's duct was apparent on T1-weighted imaging (Fig. 3C). T2-weighted imaging showed multiple spots of high signal intensity within the left parotid gland and a cystic high-signal extension at the orifice (Fig. 3D). No salivary stones were identified on MR imaging.

**Fig. 4:**
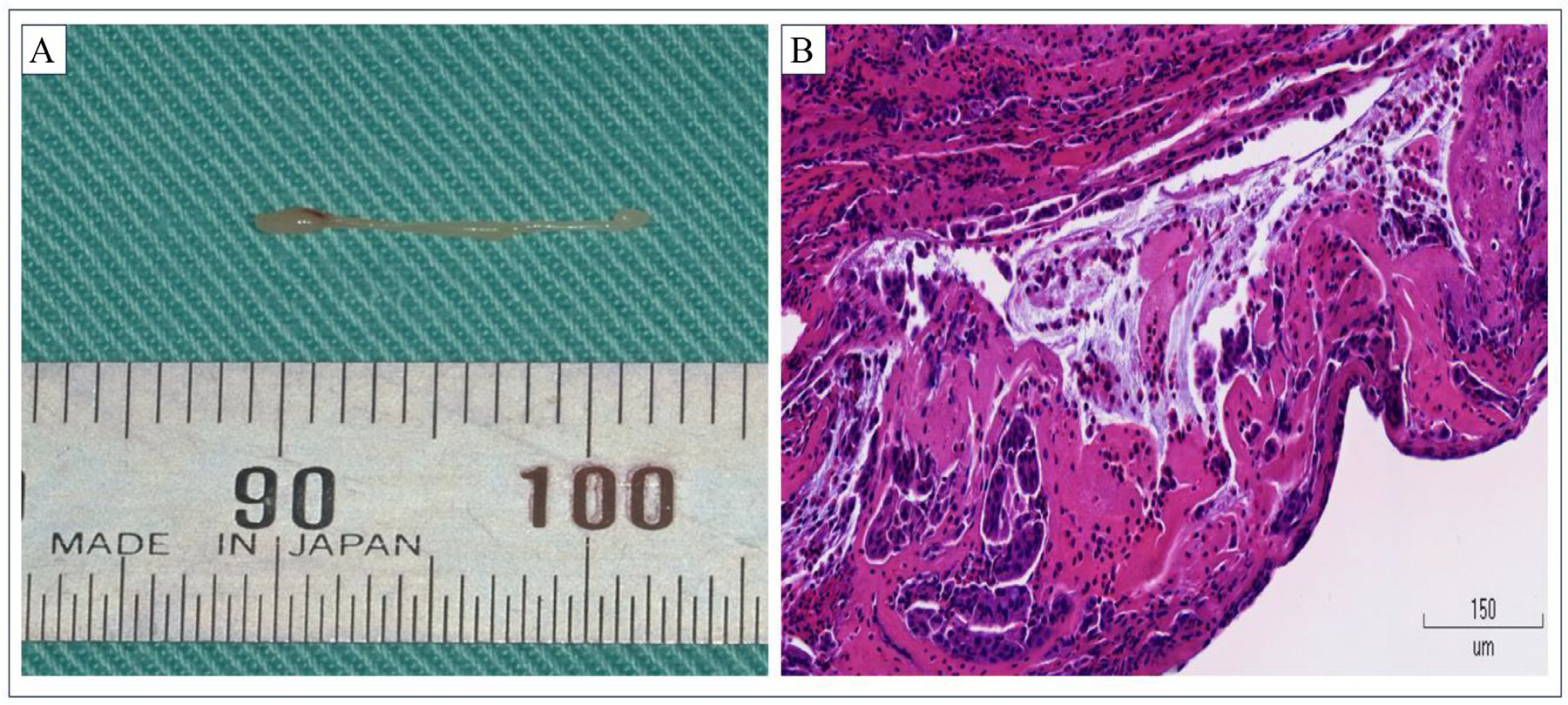
(A) Macroscopic view of a white mass 13 mm in length that was discharged into the oral cavity through the left Stensen's duct. (B) Microscopic view with hematoxylin and eosin staining of discharged tissue. Numerous eosinophils and fragments of degenerated ductal epithelia are seen against a background of eosinophilic fibrin.

Histopathological analysis of a white fibrous mass 13 mm in length that was discharged from the parotid papilla revealed that the mass comprised inflammatory exudate with prominent eosinophil infiltration and numerous fragments of ductal epithelia (Fig. 4).

Blood test results were as follows. Eosinophils were high (13.9%), anti-SS-A and anti-SS-B antibodies were negative, and radioallergosorbent test (RAST) showed elevated levels of house dust, house mite, cat, and dog allergens. There were no other abnormalities. Based on the above clinical and imaging findings, a diagnosis of sialodochitis fibrinosa was made.

Treatment included compressive massage of the glandular body in the parotid region, bougie enlargement of the stenosis in the orifice, flushing of the duct, and systemic antihistamine administration. The antihistamine was changed to long-acting rupatadine after improvement was seen with desloratadine. However, the patient became drowsy during the day, and the medication was changed to bepotastine, which is fast acting. At the last follow-up visit, the patient reported some continued discomfort but no white discharge or itching.

## Discussion

The present patient had prominent swelling of the left parotid gland and discharge of white mass material into the oral cavity. The imaging findings were characterized as multiple cystic dilations within the left parotid gland and cystic dilation due to stenosis of the orifice of the left Stensen's duct. Treatment included compressive massage of the body of the parotid gland, bougie enlargement of the stenotic orifice, flushing of Stensen's duct, and systemic antihistamine administration, which achieved a satisfactory outcome.

Sialodochitis fibrinosa was first described by Kussmaul in 1897 as a repetitive and paroxysmal swelling of the salivary glands caused by obstruction of the excretory duct by fibrous masses [1]. It occurs predominantly in the parotid gland, but can also occur in the submandibular gland, or in both. It is more commonly bilateral but may also be unilateral [4].

Murakami et al. have reported the characteristics of sialodochitis fibrinosa, but no definitive diagnostic criteria have yet been published [2]. Kato et al. differentiated it from other diseases based on the presence of main duct dilation [5]. However, as in the present case, lesions may also be present in the glandular body. Therefore, the diagnosis should consider clinical symptoms such as fibrous white matter discharge and increased eosinophils in the discharge as well as the imaging findings. As this disease is commonly associated with allergic disorders, it has been considered a type of allergic parotitis [10]. Inflammatory lesions of the salivary glands include Sjögren's syndrome, Kimura's disease, and IgG4-related diseases [11].

Sialography, MRI, ultrasonography, and CT are useful in the diagnosis of salivary gland disease. In a previous study of Japanese patients, sialodochitis fibrinosa was characterized by occasional focal stenosis of the salivary gland ducts, indistinct gland shadows, irregular dilation of main salivary duct, leakage of contrast material from main salivary duct, and apparently normal images [3]. To the best of our knowledge, 32 cases of sialodochitis fibrinosa have been reported, 17 of which underwent sialography. Dilation of the salivary gland ducts, generally of the main duct, was common, but dilation of the orifice, as in the present patient, was found in only 1 case [6]. The present patient underwent STIR imaging rather than sialography, and this is the first report of the utility of STIR for assessment of sialodochitis fibrinosa. In our patient, STIR depicted multiple cystic dilations within the left parotid gland body, cystic dilation near the orifice, and dilation of Stensen's duct. Of 13 patients who underwent MRI for evaluation of sialodochitis fibrinosa in previous studies, the main duct was dilated in all cases, and multiple cystic dilations were seen within the glandular body in 4 cases [6,7,8]. Although MRI has lower resolution than sialography, it is noninvasive and can visualize multiple areas including the glandular body to determine the exact location of a lesion in cross-sectional images. Importantly, dilation of the left lateral Stensen's duct orifice (which is apparent on T1-weighted imaging) and multiple high-signal-intensity spots within the left parotid gland body and cystic high-signal dilation near the orifice (which are apparent on T2-weighted imaging) can both be assessed on STIR images. This may be because fat suppression with the STIR sequence is relatively unaffected by magnetic field inhomogeneities and has low distortion.

In the present patient, the choice of treatment was based on the imaging findings. Compressive massage was performed in the body of the gland, bougie enlargement was performed on the stenosis at the orifice, duct flushing was performed on Stensen's duct, and systemic drugs were administered. Of the 2 main treatment strategies, conservative treatment and surgical treatment, conservative treatment includes medication, local cleansing, and physical treatment, often in combination. Regarding medications, steroids and antihistamines are generally prescribed due to the high incidence of allergic complications. However, several cases of relapse have been reported after switching to antihistamines to avoid the side effects of long- term steroid administration [9,12]. Of a total of 22 previously reported cases that received conservative treatment alone, only 3 were completely cured [6,7,13]. In these 22 previous cases, 3 of 4 cases in which lesions were identified in the glandular body by imaging were treated with drugs alone and 1 was treated with drugs and parotid massage. In all 4 cases, treatment ameliorated the symptoms to some degree; however, the symptoms were controlled by medication alone in a patient with dilated orifice.

Surgical treatment is the first choice for complete cure of sialodochitis fibrinosa, but discharge of white material from the orifice of Stensen's duct can continue after removal of the glandular body alone [14]. In other cases, removal of the bilateral Walton's ducts has been effective [15]. These cases suggest that the lesion is located within the glandular duct. However, as in our case, the lesion may be located within the glandular body, so it is necessary to confirm by imaging whether the lesion is within the gland or the glandular duct. MRI appears to be an appropriate modality for confirmation. It is important to avoid highly invasive procedures such as excision of both glandular bodies and the glandular ducts because surgical treatment is highly invasive. Surgery should be reserved for patients who have a history of chronic symptoms that have shown no improvement with conservative treatment alone.

In summary, treatment in the present case was selected according to MR findings of the parotid gland, the orifice, and Stensen's duct. Parotid massage was chosen for treatment of multiple cystic dilations in the gland, bougie enlargement for cystic dilation due to stenosis of the orifice, and duct flushing for dilation of the duct, which were successful. The MRI findings were comparable to those of sialography, enabled observation of multiple areas, and provided cross-sectional images. In particular, STIR imaging allowed detailed observation of the site of onset. Therefore, MRI is a suitable noninvasive alternative to sialography. MRI can clarify the site of onset, which is important in deciding the most appropriate treatment method and is valuable in refractory cases when choosing the treatment and planning surgical procedures.

## Author contributions

All authors meet the ICMJE authorship criteria.

## Ethics approval

All procedures complied with the ethical standards of the Tokyo Dental College and with the Helsinki Declaration of 1975, as revised in 2008 (5).

## Consent for publication

The patient provided written informed consent for publication of this case report and the accompanying images.

## Patient consent

The patient provided written informed consent for the treatment described in this case report.
